# Supplementary material for: A systematic review of the untreated mortality of murine typhus
Source: PLoS Negl Trop Dis. 2020 Sep 14;14(9):e0008641. doi: 10.1371/journal.pntd.0008641 (PMC7515178; doi:10.1371/journal.pntd.0008641)

**Global Health June 28^th^, 2018 search results**


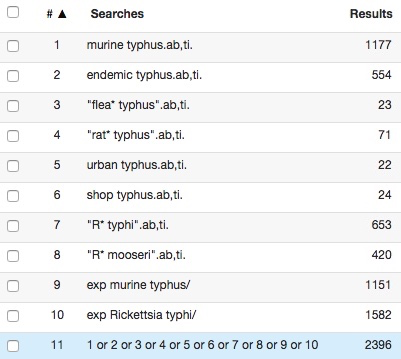


**Embase and Embase classic June 28^th^, 2018 search results**


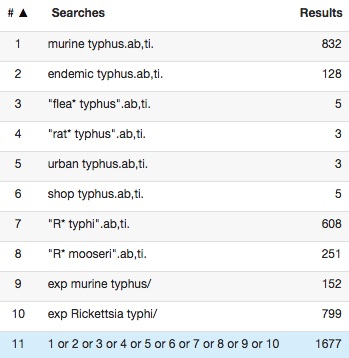


**Medline June 28^th^, 2018 search results**


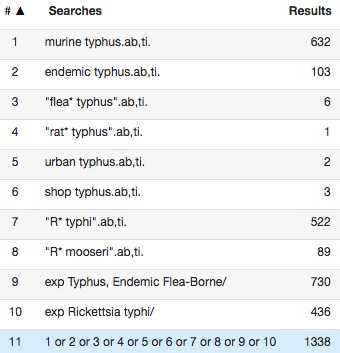

Supplement: S1 Fig — (DOCX) [file pntd.0008641.s002.docx]
