# Supplementary material for: A systematic review of the untreated mortality of murine typhus
Source: PLoS Negl Trop Dis. 2020 Sep 14;14(9):e0008641. doi: 10.1371/journal.pntd.0008641 (PMC7515178; doi:10.1371/journal.pntd.0008641)
Supplement: S1 Table — (DOCX) [file pntd.0008641.s003.docx]

**Assessment of bias form**

| Category | Grade | Criteria |
| --- | --- | --- |
| Patient selection | **I** | Prospective case series without exclusion of patients |
|  | **II** | Retrospective case series without exclusion of patients |
|  | **III** | Patients excluded |
| Diagnostic test | **I** | Molecular detection of *R. typhi* in clinical specimen  Serological detection of antibodies against *R. typhi* with differentiation from *R. prowazekii* |
|  | **II** | Serological detection of antibodies against *R. typhi* for all patients, without differentiation from *R. prowazekii* |
|  | **III** | Serological detection of antibodies against *R. typhi* for the majority of patient cases, remainder diagnosed by Weil-Felix test |
| Missing information | **I** | No missing information |
|  | **II** | Missing information on 1 or 2 of age, sex, fever duration or complications |
|  | **III** | Missing information on 3 or more of age, sex, fever duration or complications |
