# Supplementary material for: A systematic review of the untreated mortality of murine typhus
Source: PLoS Negl Trop Dis. 2020 Sep 14;14(9):e0008641. doi: 10.1371/journal.pntd.0008641 (PMC7515178; doi:10.1371/journal.pntd.0008641)
Supplement: S2 Table — (DOCX) [file pntd.0008641.s004.docx]

**Articles for which full-text was not retrievable**

| # | First author | Title | Journal |  | Year |
| --- | --- | --- | --- | --- | --- |
| 1 | C E VAN ROOYEN | Typhus Rickettsial Agglutination Tests in the M.E.P. and Egypt | Journal of the Egyptian Public Health Association |  | 1944 |
| 2 | H Y CHUNG | Studies on murine typhus in Korea | The Korean journal of internal medicine |  | 1962 |

**Articles excluded because of missing information on diagnosis, treatment or outcome**

| # | Author | Title | Journal | Year |
| --- | --- | --- | --- | --- |
| 1 | C E VAN ROOYEN | Typhus Rickettsial Agglutination Tests in the Middle East Forces and Egypt. | Edinburgh Medical Journal | 1943 |
| 2 | C R ESKEY | Murine Typhus Fever Control. | Public Health Reports | 1943 |
| 3 | H PLOTZ | Endemic Typhus Fever in Jamaica, B.W.I. | American Journal of Public Health | 1943 |
| 4 | C E VAN ROOYEN | Typhus Research in Egypt, Palestine, Iraq and Iran. | Transactions of the Royal Society of Tropical Medicine and Hygiene | 1944 |
| 5 | H PLOTZ | Modification of Serological Response to Infection with Murine Typhus by Previous Immunization with Epidemic Typhus Vaccine. | Proceedings of the Society for Experimental Biology and Medicine | 1945 |
| 6 | G S BOTE | The prevalence of endemic typhus fever in Florida. | Florida health notes | 1946 |
| 7 | J C DICK | Notes on the Weil-Felix Reaction in Typhus Fever and other Diseases. | J. & Bact | 1946 |
| 8 | Seaton D.R. | A serological analysis of typhus cases in India by Weil-Felix, rickettsial agglutination and complement-fixation tests | Annals of Tropical Medicine and Parasitology | 1946 |
| 9 | C T NELSON | The Serologic Response in Murine Typhus as measured by the Weil-Felix, Rickettsial Complement Fixation, and Rickettsial Agglutination Reactions. | Journal of Laboratory and Clinical Medicine | 1947 |
| 10 | D E DAVIS | The Use of DDT to control Murine Typhus Fever in San Antonio, Texas. | Public Health Reports | 1947 |
| 11 | GRAY A.L. | The epidemiology of murine typhus in Mississippi in 1946. | The Mississippi doctor | 1947 |
| 12 | M Dorthy. BECK | Typhus Fever in California, 1916-1945, Inclusive. An Epidemiologic and Field Laboratory Study. | American Journal of Hygiene | 1947 |
| 13 | M E BUSTAMANTE | Distribution of Rickettsial Disease in Mexico. (Murine and Epidemic Typhus and Rocky Mountain Spotted Fever.). | Revista del Instituto de Salubridad y Enfermedades Tropicales | 1947 |
| 14 | PETERSON J.C | Rickettsial diseases of childhood; a clinical pathologic study of tick typhus, Rocky Mountain spotted fever and murine typhus, endemic typhus | The Journal of pediatrics | 1947 |
| 15 | R S GOYTIA | A Contribution to the Study of the Erythrocyte Sedimentation Rate in Typhus Patients | Revista del Instituto de Salubridad y Enfermedades Tropicales | 1947 |
| 16 | A B SCOVILLE Jr | The Serological Pattern in Typhus Fever. II. Murine. | American Journal of Hygiene | 1948 |
| 17 | A POMALES LEBRON | Studies on Murine Typhus in Puerto Rico. | Puerto Rico Journal of Public Health and Tropical Medicine | 1948 |
| 18 | E R RICKARD | Complement Fixation in Human Sera following Murine Typhus. | Proceedings of the Society for Experimental Biology and Medicine | 1948 |
| 19 | E R RICKARD | Murine Typhus Fever. | Journal of the Florida Medical Association. | 1949 |
| 20 | Freeman G. | Typhus fever in Mexico; a study of epidemiology by means of complement-fixation | American Journal of Tropical Medicine | 1949 |
| 21 | Department of Public Health, California | Typhus Fever in California, 1916-1948, Inclusive | Department of Public Health California | 1950 |
| 22 | De Magalhaes O. | A subsidiary study on the diagnosis of diseases of the exanthematic typhus group in Brazil | Mem. Inst. Osw. Cruz. | 1950 |
| 23 | S CASTORINA | Complications of murine typhus | Riforma medica | 1950 |
| 24 | V SCAFFIDI | Knowledge and problems of exanthematous typhus of murin origin | Acta Medica Italica di Malattie Infettive e Parassitarie | 1950 |
| 25 | V SCAFFIDI | Symptoms and complications of the nervous system in murine typhus | Riforma medica | 1950 |
| 26 | Baird R.B. | Endemic typhus in Mengo district, Uganda | East African medical journal | 1951 |
| 27 | J C HEDDON | Murine typhus fever; its incidence and control in South Carolina. | Journal of the South Carolina Medical Association | 1951 |
| 28 | Kalra S.L. | Typhus in Bangalore and Mysore | Indian Journal of Medical Research | 1951 |
| 29 | Kalra S.L. | Typhus fevers in Kashmir State. II. Murine typhus | Indian Journal of Medical Research | 1951 |
| 30 | M KITAOKA | On Epidemiology of Murine Typhus in Saitama 1948, in Niigata 1949 and Multiple Occurrence of Murine Typhus in one Family. | Japanese Medical Journal | 1951 |
| 31 | P M RODRIGUES | The Bengtson (Complement-Fixation) Test in the Diagnosis of Benign Rickettsioses in Sao Paulo. | Revista do Instituto Adolfo Lutz | 1951 |
| 32 | Jean. JADIN | The Rickettsial Diseases of the Belgian Congo and Ruanda-Urundi. | Annales de l'Institut Pasteur | 1952 |
| 33 | V KNIGHT | Treatment of endemic and epidemic typhus with antibiotics. | Annals of the New York Academy of Sciences | 1952 |
| 34 | Quinby G.E. | Epidemiologic and serologic appraisal of murine typhus in the United States, 1948-1951 | Amer. J. Publ. Hlth. | 1953 |
| 35 | A ANADAO | The high incidence of murine typhus in the town of Sao Sebastiao da Grama (Sao Paulo) | Revista Paulista de Medicina | 1954 |
| 36 | Stewart W.H. | Murine Typhus Fever in South-West Georgia, January 1945-January 1953. | Amer. J. Trop. Med. | 1954 |
| 37 | P SUREAU | Murine Typhus at Dalat, South Vietnam; the Present Situation. Isolation of a Strain. | Bulletin de la Societe de Pathologie Exotique | 1955 |
| 38 | D F HERSEY | Studies on the Serologie Diagnosis of Murine Typhus and Rooky Mountain Spotted Fever. II. Human Infections. | Journal of Immunology | 1957 |
| 39 | S A IMAMALIEV | Clinical and Epidemiological Characteristics of Endemic (Rat) Exanthematic Typhus. | Journal of Microbiology, Epidemiology and Immunobiology | 1957 |
| 40 | G VARELA | Serological Study of the Distribution of Typhus in the Mexican Republic in 1958. | Bol. Epidemiologico | 1959 |
| 41 | E H DERRICK | Murine Typhus, Mice, Rats and Fleas on the Darling Downs. | Medical Journal of Australia | 1960 |
| 42 | A G SOMOVA | On the problem of endemic rat typhus fever on the Black Sea coast | Zhurnal mikrobiologii, epidemiologii, i immunobiologii | 1960 |
| 43 | T C WENG | Clinical observation on two weeks fever (murine typhus) in Taiwan | Journal of the Formosan Medical Association | 1961 |
| 44 | I Z E IMAM | Murine Typhus in Egypt U.A.R. | Journal of the Egyptian Public Health Association | 1962 |
| 45 | D BEYTOUT | Demonstration of Rickettsial Diseases by means of the Microagglutination Test at Saigon, 1962-1963. | Bulletin de la Societe de Pathologie Exotique | 1964 |
| 46 | H PEQUIGNOT | Rickettsial diseases observed in France | Revue du Praticien | 1964 |
| 47 | Luis Vassallo | Murine Typhus in the Maltese Islands | Annals of Tropical Medicine & Parasitology | 1970 |
| 48 | J J Older | Epidemiology of Rocky Mountain spotted fever and murine typhus | Texas medicine | 1970 |
| 49 | V URLIC | Endemic murine typhus in Dalmatia. Biological and epidemiological studies of a focus with an endemic character | Giornale di Malattie Infettive e Parassitarie | 1971 |
| 50 | Miller M.B. | Murine typhus in Vietnam. | Military Medicine | 1974 |
| 51 | T Rosenthal | Murine typhus and spotted fever in Israel in the seventies. | Infection | 1977 |
| 52 | Al-Awadi A.R | Murine typhus in Kuwait in 1978 | Bulletin of the World Health Organization | 1982 |
| 53 | ANONYMOUS | Investigations on an outbreak of murine typhus fever | Zhonghua liu xing bing xue za zhi = Zhonghua liuxingbingxue zazhi | 1983 |
| 54 | Jeffery P. Taylor | Epidemiology of Murine Typhus in Texas: 1980 Through 1984 | JAMA: The Journal of the American Medical Association | 1986 |
| 55 | T E Woodward | Keep murine typhus in mind | Journal of the American Medical Association | 1986 |
| 56 | Y Tselentis | An endemic focus of murine typhus in Europe | Journal of Infection | 1986 |
| 57 | C H Won | Seroepidemiologic study of acute hemorrhagic diseases in Korea, 1989. | Korea University Medical Journal | 1991 |
| 58 | Chayakul P | Murine typhus in Thailand: clinical features, diagnosis and treatment | Quarterly Journal of Medicine | 1993 |
| 59 | F. J. Sorvillo | A suburban focus of endemic typhus in Los Angeles County: Association with seropositive domestic cats and opossums | American Journal of Tropical Medicine and Hygiene | 1993 |
| 60 | Silpapojakul K | Liver involvement in murine typhus. | QJM : monthly journal of the Association of Physicians | 1996 |
| 61 | G Perez-Avraham | Zoonotic infections as causes of hospitalization among febrile Bedouin patients in southern Israel. | Transactions of the Royal Society of Tropical Medicine and Hygiene | 2001 |
| 62 | Ong A.K. | Endemic typhus in Singapore--a re-emerging infectious disease? | Singapore medical journal | 2001 |
| 63 | Roberts S. | Murine typhus in New Zealand | New Zealand Public Health Report | 2001 |
| 64 | J Bishara | Murine typhus among Arabs and Jews in Israel 1991-2001. | European Journal of Epidemiology | 2004 |
| 65 | C Suttinont | Causes of acute, undifferentiated, febrile illness in rural Thailand: results of a prospective observational study. | Annals of Tropical Medicine and Parasitology | 2006 |
| 66 | Phongmany S | Rickettsial infections and fever, Vientiane, Laos. | Emerging Infectious Diseases | 2006 |
| 67 | M Hidalgo | Murine typhus in Caldas, Colombia. | American Journal of Tropical Medicine and Hygiene | 2008 |
| 68 | Mark D. Zimmerman | Murine typhus and febrile illness, Nepal | Emerging Infectious Diseases | 2008 |
| 69 | V Punda-Polic | Epidemiological features of Mediterranean spotted fever, murine typhus, and Q fever in Split-Dalmatia County (Croatia), 1982-2002 | Epidemiology and Infection | 2008 |
| 70 | Nan-Yu Chen | Clinical prediction of endemic rickettsioses in northern Taiwan--relevance of peripheral blood atypical lymphocytes. | Journal of microbiology, immunology, and infection = Wei mian yu gan ran za zhi | 2008 |
| 71 | N Kaabia | Rickettsial infection in hospitalised patients in central Tunisia: report of 119 cases. | Clinical Microbiology and Infection | 2009 |
| 72 | Suputtamongkol Y. | Epidemiology and clinical aspects of rickettsioses in Thailand | Annals of the New York Academy of Sciences | 2009 |
| 73 | Pradhan R | Bloodstream infection among children presenting to a general hospital outpatient clinic in urban Nepal. | PLoS ONE | 2012 |
| 74 | Psaroulaki A | Murine typhus in Cyprus: a 9-year survey. | Transactions of the Royal Society of Tropical Medicine and Hygiene | 2012 |
| 75 | Anyfantakis D | Liver function test abnormalities in murine typhus in Greece: a retrospective study of 165 cases. | Le infezioni in medicina: rivista periodica di eziologia, epidemiologia, diagnostica, clinica e terapia delle patologie infettive | 2013 |
| 76 | Balleydier E | Emergence of murine typhus in Reunion Island, South West Ocean Indian Island: Epidemiological, clinical, laboratory features of 10 cases | International Journal of Infectious Diseases | 2014 |
| 77 | Murray K.O | The emergence and epidemiology of endemic (flea-borne) typhus in texas, 2003-2013 | American Journal of Tropical Medicine and Hygiene | 2016 |
| 78 | S Bhengsri | Sennetsu neorickettsiosis, spotted fever group, and typhus group rickettsioses in three provinces in Thailand. | American Journal of Tropical Medicine and Hygiene | 2016 |
| 79 | Alisjahbana B. | Rickettsial infection: An unexpected cause of fever in patients hospitalized with acute febrile illness in Indonesia | American Journal of Tropical Medicine and Hygiene | 2017 |
| 80 | Chang K | Characteristics of scrub typhus, murine typhus, and Q fever among elderly patients: Prolonged prothrombin time as a predictor for severity. | Journal of Microbiology, Immunology and Infection | 2017 |
| 81 | Murray K.O., | Typhus group Rickettsiosis, Texas, USA, 2003-2013 | Emerging Infectious Diseases | 2017 |
| 82 | Pieracci E.G. | Fatal flea-borne typhus in Texas: A retrospective case series, 1985-2015 | American Journal of Tropical Medicine and Hygiene | 2017 |
| 83 | Howard A | Murine Typhus in South Texas Children: An 18-Year Review. | The Pediatric infectious disease journal | 2018 |
| 84 | Kingston H.W | Rickettsial Illnesses as Important Causes of Febrile Illness in Chittagong, Bangladesh. | Emerging infectious diseases | 2018 |
| 85 | Rahat F Vohra | Analysis of health-Care charges in murine typhus: Need for improved clinical recognition and diagnostics for acute disease | American Journal of Tropical Medicine and Hygiene | 2018 |

**Articles excluded because of uncertain diagnosis**

| # | Author | Title | Journal | Year |
| --- | --- | --- | --- | --- |
| 1 | Henry E Meleney | Recent Extension of Endemic Typhus Fever in the Southern United States. | American Journal of Public Health | 1941 |
| 2 | Ida A BENGTSON | The Specificity of the Complement Fixation Test in Endemic Typhus Fever using a Rickettsial Antigen. | Public Health Reports | 1941 |
| 3 | Ida A BENGTSON | Complement-Fixation in Rickettsial Diseases. | American Journal of Public Health | 1942 |
| 4 | Wei-T'ung Liu | Typhus Fever in Peiping. Epidemiological Considerations. | American Journal of Hygiene | 1942 |
| 5 | C G BAKER | Endemic Typhus Fever in Diego Suarez, Madagascar. | British Medical Journal | 1943 |
| 6 | Norman H TOPPING | Apparent Recent Extension of Typhus in the United States. | American Journal of Tropical Medicine | 1943 |
| 7 | H R RUGIERO | Further Cases of Exanthematic Typhus in Buenos Aires and its Environs | Prensa Medica Argentina | 1945 |
| 8 | B M STUART | Endemic (Murine) Typhus Fever : Clinical Observations of 180 Cases. | Ann. Intern. Med. | 1945 |
| 9 | G ARBONA | Atabrine in the Treatment of Endemic Typhus Fever. | Boletin de la Asociacion Medica de Puerto Rico | 1945 |
| 10 | H E MELENEY | Endemic Typhus Fever in Southern California. | California & Western Med. | 1945 |
| 11 | P K SMITH | The use of para-aminobenzoic acid in endemic (marine) typhus. | Journal of the American Medical Association | 1946 |
| 12 | E L HILL | A Study of Murine Typhus Fever In Coffee County, Alabama. | Public Health Reports | 1947 |
| 13 | J CHESNUT | Endemic typhus fever. Clinical study of twenty-four cases. Review of literature | West Virginia Medical Journal | 1947 |
| 14 | J H RAYNAL | Studies of Typhus. The Behaviour of the Disease in Shanghai from 1938 to 1945. | Medecine Tropicale | 1947 |
| 15 | C GRASSI BERTAZZI | Il chinino e la maretina nella terapia del tifo murino della Sicilia orientale; primi risultati clinici e terapeutici | Minerva Medica | 1948 |
| 16 | H FULLER | Murine typhus; treatment of a small series of cases with para-aminobenzoic acid | The Journal of the Florida Medical Association. Florida Medical Association | 1948 |
| 17 | RICKARD E.R. | A State-Wide Survey of Typhus Fever in Florida. | American Journal of Public Health | 1948 |
| 18 | Bormann F | The problem of whether an attack of typhus confers life-long immunity | Deutsche Medizinische Wochenschrift | 1950 |
| 19 | Ortiz Mariotte C. | Rickettsiosis in Urupan. Preliminary report | Medicina Revista Mexicana | 1950 |
| 20 | E G BABALOVA | Murine rickettsiosis in the city B.; clinical aspects of the disease | Zhurnal mikrobiologii, epidemiologii, i immunobiologii | 1954 |
| 21 | Jessica Rauch | Typhus Group Rickettsiosis, Germany, 2010-2017. | Emerging infectious diseases | 2018 |

**Articles excluded because of adequate treatment of patients**

| # | Author | Title | Journal | Year |
| --- | --- | --- | --- | --- |
| 1 | R LEWTHWAITE | Chloromycetin in the treatment of typhus and typhoid. | Proceedings of the Royal Society of Medicine | 1950 |
| 2 | C E BUSH | Diagnosis in the Department of Lambayeque of first cases of murine typhus in Peru | Revista Medica Peruana | 1950 |
| 3 | T BRICENO MAAZ | Murine Typhus. A Study of 33 Cases in Venezuela. | Archivos Venezolanos de Patologia Tropical y Parasitologia Medica | 1950 |
| 4 | V URLIC | Murine typhus in Dalmatia | Medicinski glasnik | 1961 |
| 5 | J J RESSELER | A study of flea-borne typhus in Kasai | Annales de la Societe belge de medecine tropicale | 1962 |
| 6 | Older J | The epidemiology of murine typhus in Texas, 1969 | Journal of the American Medical Association | 1970 |
| 7 | SANGKASUWAN V | Murine typhus. A report of 15 cases | Journal of the Medical Association of Thailand | 1973 |
| 8 | Betz T.G | Endemic typhus in Texas | Texas medicine | 1983 |
| 9 | SILPAPOJAKUL K | Murine typhus in southern Thailand | Journal of the Medical Association of Thailand | 1987 |
| 10 | Brown A.E | Murine typhus among Khmers living at an evacuation site on the Thai-Kampuchean border | American Journal of Tropical Medicine and Hygiene | 1988 |
| 11 | P E Duffy | Murine typhus identified as a major cause of febrile illness in a camp for displaced Khmers in Thailand. | American Journal of Tropical Medicine and Hygiene | 1990 |
| 12 | Chupuppakarn S. | Scrub and murine typhus in children with obscure fever in the tropics. | Pediatric Infectious Disease Journal | 1991 |
| 13 | Tselentis Y. | Clinicoepidemiological study of murine typhus on the Greek island of Evia. | European Journal of Epidemiology | 1992 |
| 14 | Shaked Y. | Involvement of the kidneys in Mediterranean spotted fever and murine typhus | Quarterly Journal of Medicine | 1994 |
| 15 | Kaabia N | Serologic study of rickettsioses among acute febrile patients in Central Tunisia | Annals of the New York Academy of Sciences | 2006 |
| 16 | Wu Jun | Clinical analysis of 83 endemic typhus cases. | China Tropical Medicine | 2009 |
| 17 | Chang K | Murine typhus in southern Taiwan during 1992-2009. | American Journal of Tropical Medicine and Hygiene | 2012 |
| 18 | G Chaliotis | Murine typhus in central Greece: epidemiological, clinical, laboratory, and therapeutic-response features of 90 cases. | International Journal of Infectious Diseases | 2012 |
| 19 | Walter G. | Murine typhus in returned travelers: a report of thirty-two cases. | American Journal of Tropical Medicine and Hygiene | 2012 |
| 20 | Yang W.-H. | Murine typhus in drug detoxification facility, Yunnan Province, China, 2010. | Emerging Infectious Diseases | 2012 |
| 21 | Aouam A. | Epidemiological, clinical and laboratory features of murine typhus in central Tunisia. | Medecine et Maladies Infectieuses | 2015 |
| 22 | Blanton L.S. | Reemergence of murine typhus in Galveston, Texas, USA, 2013. | Emerging Infectious Diseases | 2015 |
| 23 | Hamaguchi S | Clinical and epidemiological characteristics of scrub typhus and murine typhus among hospitalized patients with acute undifferentiated fever in Northern Vietnam | American Journal of Tropical Medicine and Hygiene | 2015 |
| 24 | Erickson T. | Newly recognized pediatric cases of typhus group rickettsiosis, Houston, Texas, USA | Emerging Infectious Diseases | 2017 |

**Articles excluded because untreated patients < 5**

| # | Author | Title | Journal | Year |
| --- | --- | --- | --- | --- |
| 1 | Davis J.W | Endemic typhus in Baltimore | Southern Medical Journal | 1948 |

**Articles excluded because of duplicate case series**

| # | Author | Title | Journal | Year |
| --- | --- | --- | --- | --- |
| 1 | L F Lopez Cortes | Murine typhus: clinical and serological review of 42 cases | Medicina clinica | 1983 |
| 2 | J Campbell | Outbreak of Rickettsia typhi infection - Austin, Texas, 2008. | Morbidity and Mortality Weekly Report | 2009 |

**Articles excluded because not about murine typhus**

| # | Author | Title | Journal | Year |
| --- | --- | --- | --- | --- |
| 1 | S FRANCK | Epidemic of mouse typhus | Militaerlaegen | 1956 |
