# Supplementary material for: A systematic review of the untreated mortality of murine typhus
Source: PLoS Negl Trop Dis. 2020 Sep 14;14(9):e0008641. doi: 10.1371/journal.pntd.0008641 (PMC7515178; doi:10.1371/journal.pntd.0008641)
Supplement: S3 Table — (DOCX) [file pntd.0008641.s005.docx]

**Bias assessment of included studies**

| Study | Patient selection | Diagnostic test | Missing information |
| --- | --- | --- | --- |
| Crofton 1944 | **II** | **I** | **III** |
| Calero 1948 | **I** | **III** | **I** |
| Diaz-Rivera 1949 | **III** | **II** | **II** |
| Shaked 1988 | **III** | **II** | **II** |
| Dumler 1990 | **III** | **II** | **III** |
| Bernabeu-Wittel 1999 | **III** | **II** | **III** |
| Hernández-Cabrera 2004 | **III** | **II** | **III** |
| Shalev 2006 | **III** | **II** | **III** |
| Gray 2007 | **III** | **II** | **III** |
| Adjemian 2010 | **III** | **II** | **III** |
| Znazen 2013 | **III** | **II** | **III** |
| Afzal 2017 | **III** | **II** | **III** |
