# Supplementary material for: A systematic review of the untreated mortality of murine typhus
Source: PLoS Negl Trop Dis. 2020 Sep 14;14(9):e0008641. doi: 10.1371/journal.pntd.0008641 (PMC7515178; doi:10.1371/journal.pntd.0008641)
Supplement: Attachment — Submitted filename: Letter of response.docx [file pntd.0008641.s006.docx]

Lao-Oxford-Mahosot Hospital-Wellcome Trust Research Unit

Microbiology Laboratory

Mahosot Hospital

Vientiane

Lao PDR

From: Dr. Johannes Doppler

E-Mail: j.doppler@gmail.com

To

Editors

PLOS Neglected Tropical Diseases

26^th^ June 2020

Dear Dr. Lopez,

We thank you and the reviewers for thoroughly reading our manuscript and for the expert comments and suggestions for improvement. We are very grateful to have the opportunity to resubmit a significantly revised and improved systematic review of the untreated mortality of murine typhus. On the following pages, you will find our response to the reviewers’ comments and a description of the changes made in the manuscript.

On behalf of the co-author, we thank you for your consideration of this resubmission and look forward to your response.

Sincerely,

Johannes Doppler, MD, MSc, DTM&H

Thank you very much for submitting your manuscript "A systematic review of the untreated mortality of murine typhus" for consideration at PLOS Neglected Tropical Diseases. As with all papers reviewed by the journal, your manuscript was reviewed by members of the editorial board and by several independent reviewers. In light of the reviews (below this email), we would like to invite the resubmission of a significantly-revised version that takes into account the reviewers' comments.

We cannot make any decision about publication until we have seen the revised manuscript and your response to the reviewers' comments. Your revised manuscript is also likely to be sent to reviewers for further evaluation.

When you are ready to resubmit, please upload the following:

[1] A letter containing a detailed list of your responses to the review comments and a description of the changes you have made in the manuscript. Please note while forming your response, if your article is accepted, you may have the opportunity to make the peer review history publicly available. The record will include editor decision letters (with reviews) and your responses to reviewer comments. If eligible, we will contact you to opt in or out.

[2] Two versions of the revised manuscript: one with either highlights or tracked changes denoting where the text has been changed; the other a clean version (uploaded as the manuscript file).

Important additional instructions are given below your reviewer comments.

Please prepare and submit your revised manuscript within 60 days. If you anticipate any delay, please let us know the expected resubmission date by replying to this email. Please note that revised manuscripts received after the 60-day due date may require evaluation and peer review similar to newly submitted manuscripts.

Thank you again for your submission. We hope that our editorial process has been constructive so far, and we welcome your feedback at any time. Please don't hesitate to contact us if you have any questions or comments.

Sincerely,

Job E Lopez, Ph.D.

Deputy Editor

PLOS Neglected Tropical Diseases

Job Lopez

Deputy Editor

PLOS Neglected Tropical Diseases

***********************

Reviewer's Responses to Questions

Key Review Criteria Required for Acceptance?

As you describe the new analyses required for acceptance, please consider the following:

Methods

-Are the objectives of the study clearly articulated with a clear testable hypothesis stated?

-Is the study design appropriate to address the stated objectives?

-Is the population clearly described and appropriate for the hypothesis being tested?

-Is the sample size sufficient to ensure adequate power to address the hypothesis being tested?

-Were correct statistical analysis used to support conclusions?

-Are there concerns about ethical or regulatory requirements being met?

Reviewer #1: The applied methods are adequate and available in the manuscript and its supplements.

I would like to raise the following points to be either discussed or modified:

1. A critical point is the reliable differentiation of R. typhi cases from R. prowazekii. The exclusion of studies only based on Weil-Felix is correct. Still the serological diagnosis of these two species is very difficult and requires techniques (e.g. western blot and cross-absorption) that are only available in few reference centers in the world (see Ref 7). Epidemiological criteria might be applied, since louse-borne typhus is not widely endemic, although, especially in older studies, there might be confusion with isolated Brill-Zinser cases. These problems are briefly mentioned in Conclusions, but could be placed with a bit more information in Discussion. I think, the CF test in the 40ie was an advance, but until today the serological separation of the 2 species remains a challenge.

Thanks for raising this crucial issue. We updated the manuscript to discuss more strongly the diagnostic uncertainties of serological assays in the Introduction (Lines 89-92) and with more detail in the Discussion (Lines 384-389). We also mentioned Brill-Zinsser disease in the Discussion as a possible confounding factor in older studies (Lines 437-439).

2. The study only found a very limited number of studies and patients. Therefore every included study/patient might add valuable information. The authors excluded studies with <10 untreated patients for selection bias. I would suggest to apply this to the total number of patients (treated + untreated). In a larger series of patients with few untreated cases, I do not see selection bias problems. This approach could help to include some of the 8 studies excluded for this reason.

Thank you for this useful suggestion. We agreed to update the eligibility criteria (Lines 106-107) to include studies with ≥ 5 untreated patients and ≥ 10 treated + untreated patients, which resulted in the inclusion of an additional 5 of the 8 studies that were excluded for having less than 10 untreated patients. See Tab. 1 in the manuscript for more details on the additionally included studies.

3. Did the authors try to contact the respective author team of the newer publications to get some of the missing data?

We didn’t contact any of the respective author teams since all of the originally included papers were more than 10 years old and we thought this would unlikely be successful.

Reviewer #2: (No Response)

--------------------

Results

-Does the analysis presented match the analysis plan?

-Are the results clearly and completely presented?

-Are the figures (Tables, Images) of sufficient quality for clarity?

Reviewer #1: The results are clear and adequate.

One suggestion regarding Fig 2: due to the limited number of the included studies, the geographical "distribution" is rather random and not representative. The maps, although well prepared, are therefore of limited value and can be erased.

Since we were able to include more studies (see answer to Reviewer #1: Question 2) from different geographic regions we felt it is best to keep Fig. 2 as it provides useful visual information.

Reviewer #2: (No Response)

--------------------

Conclusions

-Are the conclusions supported by the data presented?

-Are the limitations of analysis clearly described?

-Do the authors discuss how these data can be helpful to advance our understanding of the topic under study?

-Is public health relevance addressed?

Reviewer #1: L 248: Is there evidence from treated pediatric cases that confirm this hypothesis?

We are not aware of any firm data but we cited a paper (Whiteford et al. Arch Pediatr Adolesc Med 2001;155:396-400) in the Discussion section that speculated about this hypothesis (Lines 252-254).

Discussion of diagnostic methods: see Methods

Reviewer #2: (No Response)

Editorial and Data Presentation Modifications?

Use this section for editorial suggestions as well as relatively minor modifications of existing data that would enhance clarity. If the only modifications needed are minor and/or editorial, you may wish to recommend “Minor Revision” or “Accept”.

Reviewer #1: (No Response)

Reviewer #2: (No Response)

Summary and General Comments

Use this section to provide overall comments, discuss strengths/weaknesses of the study, novelty, significance, general execution and scholarship. You may also include additional comments for the author, including concerns about dual publication, research ethics, or publication ethics. If requesting major revision, please articulate the new experiments that are needed.

Reviewer #1: Well conducted study and well written manuscript.

Therefore, only minor comments and suggestions.

Reviewer #2: The manuscript by Doppler and Newton describe an analysis of the published literature to determine the untreated case fatality rate of those with murine typhus. In general, the manuscript is well written and attempts to clarify an important question. Below are specific comments for the authors’ consideration.

Major comments:

Line 86 – 89 and throughout: The authors aim to examine literature that excludes those with epidemic typhus (R. prowazekii) from those with murine typhus (R. typhi). The former has a much higher case fatality. Considering that an antibody response developed to one typhus group species is reactive to heterologous typhus group antigen, it is unclear how even serologic techniques, unless used with cross absorption, can differentiate between epidemic typhus and murine typhus. Thus, the method to exclude those with possible R. prowazekii infection is not entirely sound. Considering the low case fatality described here, I have no doubt that the cited articles are of those with murine typhus, but the limitation of even contemporary serologic assays, and the limitations as they apply here, should be more adequately discussed.

We are thankful for raising this important issue and updated the manuscript to discuss this matter more strongly. See answer to Reviewer #1 Question 1

Eligibility criteria: There is a wide variety of criteria used by various clinicians/investigators which are considered diagnostic. Can more detail be used to describe what was considered diagnostic (e.g., seroconversion, four-fold increase in titer, single antibody titers)? Although some of this detail is listed in table 1, it does not appear complete.

We updated Table 1 to cover the diagnostic details for each of the included studies as complete as possible. Unfortunately, in some of the papers titers were not specified.

Line 155: The majority of the patients in this series were hospital based. The case fatality from this study is 0.5%. In another study conducted by reviewing the hospital records of those with murine typhus (Dumler et al. JAMA. 1991;266:1365-70), 3.8% died despite treatment. Although the case series did not review cases in which hospital records where not available (many presumed by this reviewer to be outpatients with less severe disease), this hospital based mortality is quite different than what is reported here, despite the overwhelming majority receiving treatment. In another study reviewing cases over a 30-year period in Texas (a time period when effective antibiotics were available), the case fatality was reported to be 0.4% - close to what is reported here (Pieracci et al. AJTMH. 2017;96:1088-93). These differences and possible reasons for these differences in treated vs untreated case fatality should be discussed.

Thank you for this interesting remark. We updated the manuscript to discuss differences in case fatalities in other studies and possible reasons for these differences (Lines 446-454). We also added information on the rate of hospitalised patients to Table 1 for each of the included studies.

Line 296 – 298: Although the highly cited review mentioned here may quote case fatalities of 1% and 4% in treated and untreated, respectively, other literature clearly states differently. As mentioned above, recommend discussing the case fatality rates described in other studies.

See answer above.

Minor comments:

Lines 77-82: The sentence here is a bit long and unwieldy. Suggest modifying to make more clear.

The sentence has been split into two sentences (Lines 76-81) to add clarity.

Line 128: It is unclear what “patient characteristics” means here. Can the authors clarify in the manuscript? As written, it sounds almost like demographic data, which isn’t really a possible outcome.

We changed “reported complications and patient characteristics” to “clinical features and complications” to be more precise (Line 130).

Lines 204 – 209: The publication by Crofton was said to be on the British Military. Could this have skewed the sex distribution?

Thanks for pointing this out. We added this possibility of skewing the sex distribution in the Results section (Lines 214-216)

Lines 227 – 233: Much of the discussion is repetitious with other sections of the manuscript. Suggest streamlining repetitive material in favor of including more discussion to compare and contrast the case fatality here as that reported by others.

We streamlined the discussion and deleted repetitive material in favor of discussion of differences in reported case fatalities in other studies and more detailed information on diagnostic uncertainties of serological assays.

Conclusions: Recommend consolidating the conclusion to a briefer form to emphasize key points. As written, it seems to almost be a rehash of the discussion section.

We removed repetitive material from the conclusion section.

--------------------

PLOS authors have the option to publish the peer review history of their article (what does this mean?). If published, this will include your full peer review and any attached files.

If you choose “no”, your identity will remain anonymous but your review may still be made public.

Do you want your identity to be public for this peer review? For information about this choice, including consent withdrawal, please see our Privacy Policy.

Reviewer #1: No

Reviewer #2: No

Figure Files:

While revising your submission, please upload your figure files to the Preflight Analysis and Conversion Engine (PACE) digital diagnostic tool, https://pacev2.apexcovantage.com. PACE helps ensure that figures meet PLOS requirements. To use PACE, you must first register as a user. Then, login and navigate to the UPLOAD tab, where you will find detailed instructions on how to use the tool. If you encounter any issues or have any questions when using PACE, please email us at figures@plos.org.

Data Requirements:

Please note that, as a condition of publication, PLOS' data policy requires that you make available all data used to draw the conclusions outlined in your manuscript. Data must be deposited in an appropriate repository, included within the body of the manuscript, or uploaded as supporting information. This includes all numerical values that were used to generate graphs, histograms etc.. For an example see here: http://www.plosbiology.org/article/info%3Adoi%2F10.1371%2Fjournal.pbio.1001908#s5.

Reproducibility:

To enhance the reproducibility of your results, PLOS recommends that you deposit laboratory protocols in protocols.io, where a protocol can be assigned its own identifier (DOI) such that it can be cited independently in the future. For instructions see https://journals.plos.org/plosntds/s/submission-guidelines#loc-methods

__________________________________________________

In compliance with data protection regulations, you may request that we remove your personal registration details at any time. (Use the following URL: https://www.editorialmanager.com/pntd/login.asp?a=r). Please contact the publication office if you have any questions.
